# *IDH1/2* but not *DNMT3A* mutations are suitable targets for minimal residual disease monitoring in acute myeloid leukemia patients: a study by the Acute Leukemia French Association

**DOI:** 10.18632/oncotarget.5645

**Published:** 2015-10-12

**Authors:** Houria Debarri, Delphine Lebon, Christophe Roumier, Meyling Cheok, Alice Marceau-Renaut, Olivier Nibourel, Sandrine Geffroy, Nathalie Helevaut, Philippe Rousselot, Bérengère Gruson, Claude Gardin, Marie-Lorraine Chretien, Shéhérazade Sebda, Martin Figeac, Céline Berthon, Bruno Quesnel, Nicolas Boissel, Sylvie Castaigne, Hervé Dombret, Aline Renneville, Claude Preudhomme

**Affiliations:** ^1^ Hematology Department, Lille University Hospital, Lille, France; ^2^ Hematology Laboratory, Biology and Pathology Center, Lille University Hospital, Lille, France; ^3^ Hematology Department, Amiens University Hospital, Amiens, France; ^4^ UMR-S 1172, Team 3, INSERM, Lille, France; ^5^ Functional Genomic Platform, Cancer Research Institute, Lille, France; ^6^ Hematology Department, Versailles Hospital, Le Chesnay, France; ^7^ Hematology Department, Avicenne Hospital, APHP, University Paris 13, Bobigny, France; ^8^ Hematology Department, CHU de Dijon - Le Bocage Hospital, Dijon, France; ^9^ Hematology Department, Saint-Louis Hospital, APHP, Paris, France

**Keywords:** acute myeloid leukemia, minimal residual disease, next-generation sequencing

## Abstract

Acute myeloid leukemia (AML) is a heterogeneous disease. Even within the same *NPM1*-mutated genetic subgroup, some patients harbor additional mutations in *FLT3, IDH1/2, DNMT3A* or *TET2*. Recent studies have shown the prognostic significance of minimal residual disease (MRD) in AML but it remains to be determined which molecular markers are the most suitable for MRD monitoring. Recent advances in next-generation sequencing (NGS) have provided the opportunity to use multiple molecular markers. In this study, we used NGS technology to assess MRD in 31 AML patients enrolled in the ALFA-0701 trial and harboring *NPM1* mutations associated to *IDH1/2* or *DNMT3A* mutations. *NPM1* mutation-based MRD monitoring was performed by RTqPCR. *IDH1/2* and *DNMT3A* mutations were quantified by NGS using an Ion Torrent Proton instrument with high coverage (2 million reads per sample). The monitoringof *IDH1/2* mutations showed that these mutations were reliable MRD markers that allowed the prediction of relapse in the majority of patients. Moreover, *IDH1/2* mutation status predicted relapse or disease evolution in 100% of cases if we included the patient who developed myelodysplastic syndrome. In contrast, *DNMT3A* mutations were not correlated to the disease status, as we found that a preleukemic clone with *DNMT3A* mutation persisted in 40% of the patients who were in complete remission, reflecting the persistence of clonal hematopoiesis.

## INTRODUCTION

Acute myeloid leukemia (AML) is a highly heterogeneous malignancy, especially in terms of the molecular and phenotypic characteristics. Heterogeneity is also observed within the same genetic subgroup of AML tumors. For example, within nucleophosmin 1 (*NPM1*) mutant subgroup, some patients have concomitant mutations in fms-like-*tyrosine kinase 3* (*FLT3*), isocitrate dehydrogenase (*IDH*) 1 and *2*, DNA methyltransferase 3A (*DNMT3A*) or ten-elven translocation 2 (*TET2*) genes. A multi-hit model of leukemogenesis, in which class I mutations confer the proliferation or survival advantages of blast cells and class II mutations block myeloid differentiation, has been observed in most cases of AML [1]. Recently, other studies have also reported the epigenetic effects of class III mutations on AML [2, 3]. However, the exact roles of each alteration in leukemogenesis and the mechanisms of disease progression remain largely unkown, especially with respect to recent data on molecular intraclonal heterogeneity. Recent studies have shown that minimal residual disease (MRD) in AML patients, during or after treatment, has prognostic value [4–9]. However, there are many questions regarding the clinical assessment of MRD in AML patients. First, which of the potential molecular and/or cellular markers should be assessed? Second, what type of biological sample should be analyzed? Third, where should the sensitivity threshold be set, and what are the relevant time-points to consider for MRD assessment? One study found that *IDH1/2* gene mutations persisted in patients who were in complete remission (CR), although other molecular markers were not analyzed at the time of AML diagnosis [10]. These mutations may be attributed to a preleukemic clone that acquires additional mutations promoting proliferation and differentiation block, which eventually results in leukemia. This preleukemic clone may be able to survive initial chemotherapy treatments. *DNMT3A* mutations, which occur in 20% of *de novo* AML cases, lead to abnormal DNA methylation patterns, which is likely to alter the expression of various target genes [11]. The prognostic impact of *DNMT3A* mutations seems to be unfavorable [12, 13], but their applicability MRD monitoring remains unclear [14].

Recent technological advances in next-generation sequencing (NGS) have provided new opportunities for MRD monitoring in AML patients and the possibility to simultaneously analyze multiple biomarkers and to detect subclonal populations.

In this study, we used NGS technology to monitor MRD using *IDH1/2* and *DNMT3A* mutations in a cohort of *NPM1* mutated AML patients. Our objective was to evaluate the suitability of *IDH1/2* and *DNMT3A* mutations as a target for MRD detection by NGS and to compare the data with *NPM1* mutation-based MRD assessed by RTqPCR

## RESULTS

Of the 31 *NPM1* mutated AML patients, 8 patients harbored an *IDH1* mutation, 9 an *IDH2* mutation and 15 *DNMT3A* mutation. Sequencing data showed sufficient sequencing depth with a median of 2,012,459 reads for *IDH1/2* (range: 102,657 to 5,160,118 reads) and a median of 966,298 reads for *DNMT3A* (range: 565,152 to 2,700,349 reads). This coverage allowed the detection of MRD with a sensitivity of approximately 0.001%. Despite such an extensive coverage, a median of 520 reads were positive for mutations in the negative controls, reflecting cross-contamination due to the multiple steps involved in the preparation of the gene libraries (i.e., in the intra-run steps, including preliminary PCR, barcode purity, and adaptors/barcodes ligation, and the inter-run steps, including OT2 and clonal amplification). Thus, the detection limit was 0.07% for *IDH1/2* mutation analysis (0.002 - 0.097, *p* < 0.01, Fisher's exact test) and 0.11% for *DNMT3A* mutation analysis (0.001–0.426, *p* < 0,01, Fisher's exact test).

**Figure 1:**
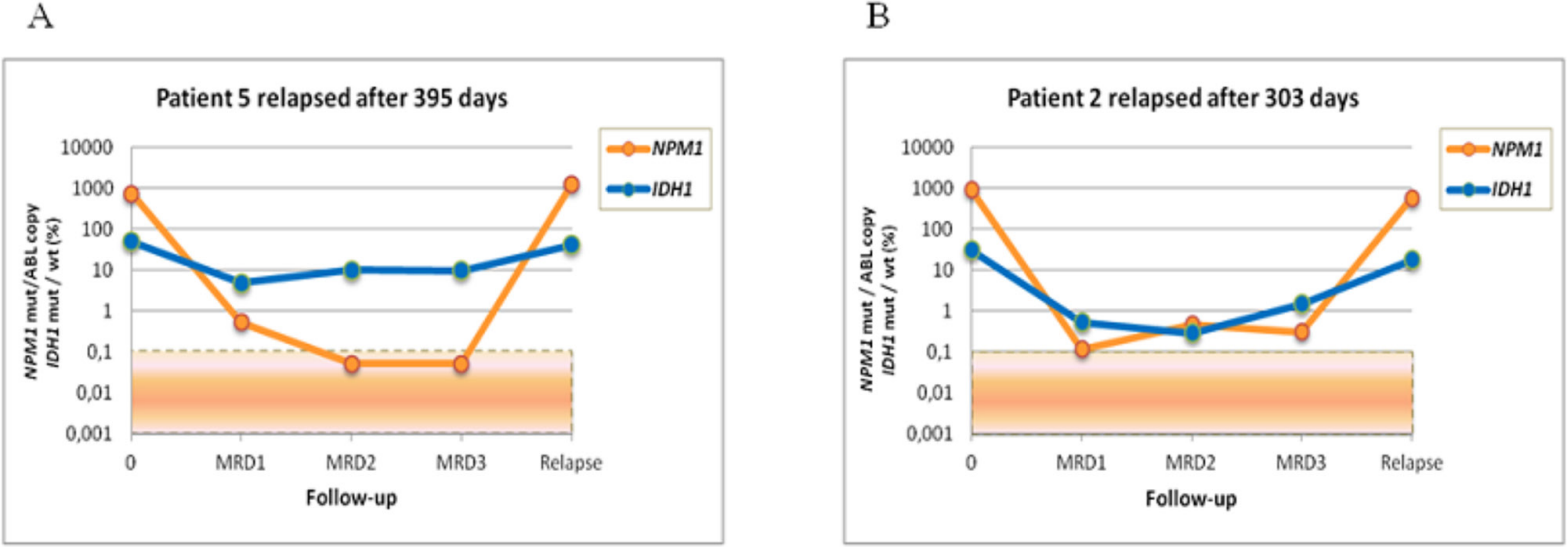
MRD monitoring in AML patients using NGS to analyze *IDH1* mutations and using RTqPCR to analyze *NPM1* mutations **A.** Discrepancy between *IDH1* and *NPM1* mutations according to the MRD stages in patient 5. **B.** Correlation between *IDH1* and *NPM1* mutations according to the MRD stages in patient 2.

**Figure 2:**
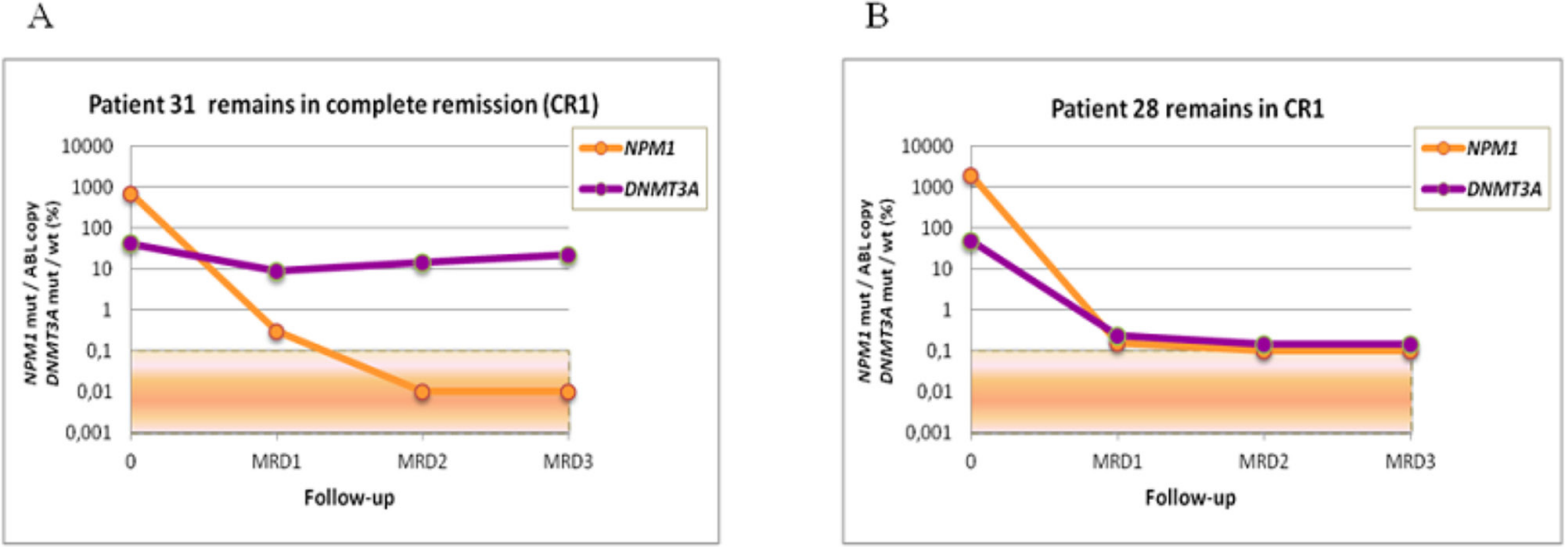
MRD monitoring in AML patients with *DNMT3A* mutations using NGS and *NPM1* mutations using quantitative RTqPCR **A.** Discrepancy between *DNMT3A* and *NPM1* mutation rates according to MRD stages in patient 31. **B.** Correlation between *DNMT3A* and *NPM1* mutations rates according to MRD stages in patient 28.

MRD level was evaluated at the following time points: post induction (MRD1), post first consolidation course (MRD2) and post second consolidation courses (MRD3). The median clinical followup of the cohort was 673 days (range: 131–2637 days). *NPM1* mutation and *IDH1/2* mutation MRD levels and *NPM1* mutation and *DNMT3A* mutation MRD levels were highly correlated (*r* = 0,68183, *p* < 0,0001; *r* = 0,55514, *p* < 0,0001, respectively). Of the 17 *IDH1/2* mutation-positive patients, we found concordant MRD results between *IDH1/2* and *NPM1* mutation levels by RTqPCR in 13 cases. Four patients who relapsed were positive for both *IDH1/2* and *NPM1* mutations, and 9 patients who remained in complete remission were negative for both *NPM1* and *IDH1/2* mutations. In the 4 remaining patients, we observed a discrepancy between the *NPM1* and *IDH1/2* mutation levels. These patients presented with one or more MRD time-points with undetectable *NPM1* mutation, whereas the *IDH1/2* mutation levels ranged from 0.5% to 47% (Table 1). Three of the patients relapsed after 504, 395 and 158 days, and all of these patients harbored similar *NPM1* mutation levels at diagnosis (Figure 1). The patient who did not relapse developed a *NPM1*-negative myelodysplastic syndrome. In the 15 *DNMT3A* mutant, we found concordant MRD results between *DNMT3A* mutation rates and *NPM1* mutation levels in 9 cases. Eight out of these 9 patients were positive for both *NPM1* and *DNMT3A* mutations and relapsed, while the remaining case, who remained in persistent CR, was negative for both *NPM1* and *DNMT3A* mutations. In the 6 discordant cases, *NPM1* mutations were undetectable in most MRD time-points, whereas *DNMT3A* mutation levels ranged from 5% to 45% during MRD follow-up. All of these patients remained in first complete remission after a median follow-up of 4 years (Table 2). *DNMT3A* mutations were detected at different time- points during follow-up (i.e., post-induction, post consolidation 1, and later), whereas other markers studied were undetectable (Figure 2). These results are consistent with clonal heterogeneity in AML, particularly on the molecular level. Samples from 3 of these patients were further examined to investigate the origin of the discrepancy using cell sorting analysis.

**Table 1:** NGS results for the 4 AML patients with discordant MRD levels between *IDH1/IDH2* mutations and *NPM1* mutation

Patients	Age	Time-point	*IDH1* R132 wild type (wt)	*IDH1* R132C mutated	%*IDH1* R132C mutated	% RTqPCR *NPM1*	Status
Patient 4	60	diagnosis	1221691	918285	**42.91**	**636**	Relapse 504 days after diagnosis
		post induction (MRD1)	2178994	152743	**6.55**	**0.13**	
		post consolidation 1 (MRD2)	2165327	202244	**8.54**	**0.02**	
Patient 8	61	diagnosis	684823	600112	**46.70**	**2155**	Relapse 158 days after diagnosis
		MRD2	1236604	765254	**38.22**	**0.01**	

Patients	Age	Time-point	*IDH1* R132 wt	*IDH1* R132H mutated	%*IDH1* R132H mutated	%RTqPCR *NPM1*	Status
Patient 5	62	diagnosis	976395	929710	**48.77**	**734**	Relapse 395 days after diagnosis
		MRD1	1712656	84303	**4.69**	**0.52**	
		MRD2	1546662	166760	**9.73**	**0.01**	
		post consolidation 2 (MRD3)	1377611	147303	**9.65**	**0.01**	
		relapse	1822544	1249478	**40.67**	**1252**	

Patients	Age	Time-point	*IDH2* R140 wt	*IDH2* R140Q mutated	%*IDH2* R140Q mutated	%RTqPCR *NPM1*	Status
Patient 17	54	diagnosis	512569	372370	**42.07**	**425.07**	Evolution to myelodysplastic syndrome
		MRD1	3848257	243415	**5.94**	**0.24**	
		MRD2	524595	473055	**47.41**	**0.01**	

**Table 2:** NGS results in the 6 AML patients who had discordant *DNMT3A* mutations compared with the results of *NPM1* mutation

Patients	Age	Time-point	*DNMT3A* R882 wild type (wt)	*DNMT3A* R882C mutated	%*DNMT3A* R882C mutated	% RTqPCR *NPM1*	Status
Patient 14	52	diagnosis	719399	619232	**46.25**	**797**	complete remission (CR) at 73 months
		post induction (MRD1)	1275523	416971	**24.63**	**0.01**	
		post consolidation 1 (MRD2)	925043	414427	**30.93**	**0.1**	
		post consolidation 2 (MRD3)	1406882	775928	**35.54**	**0.01**	
Patient 29	59	diagnosis	1596027	1284406	**44.59**	**5077.40**	CR at 41 months
		MRD1	3920773	228372	**5.50**	**0.26**	
		MRD3	3134982	579300	**15.59**	**0.01**	
Patient 31	23	diagnosis	523504	388290	**42.58**	**727.22**	CR at 47 months
		MRD1	2302664	226061	**8.93**	**0.30**	
		MRD2	2326575	391473	**14.40**	**0.01**	
		MRD3	2452633	733293	**23.01**	**0.01**	

Patients	Age	Time-point	*DNMT3A* R882 wt	*DNMT3A* R882H mutated	%*DNMT3A* R882H mutated	%RTqPCR *NPM1*	Status
Patient 20	57	diagnosis	677880	446557	**39.71**	**284**	CR at 87 months
		MRD1	787783	341300	**30.23**	**0.34**	
Patient 30	56	diagnosis	1028857	681492	**39.85**	**706.29**	CR at 46 months
		MRD1	1283146	1283146	**38.46**	**0.54**	
		MRD3	1081738	1081738	**45.25**	**0.04**	

Patients	Age	Time-point	*DNMT3A* Q886 wt	*DNMT3A* Q886E mutated	%*DNMT3A* Q886E mutated	%RTqPCR *NPM1*	Status
Patient 26	62	diagnosis	821493	559728	**40.52**	**284**	CR at 49 months
		MRD2	1477600	555883	**27.33**	**0,34**	

**Figure 3:**
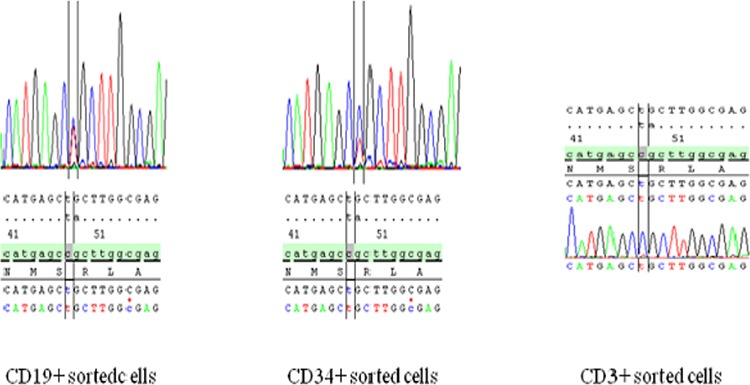
Sequencing results for the different blood fractions showing *DNMT3A* mutations in all fractions except in the CD3+ T lymphocyte fraction

In the 3 patients for which cell subpopulations were available, *DNMT3A* mutations were found in the whole peripheral blood and bone marrow collected at which time-point, but not in the DNA extracted from a skin biopsy. These findings suggest that *DNMT3A* mutations were somatically acquired (Figure 3). The percentage of mutations in which gene in these patients were comparable in all the bone marrow cell subpopulations analyzed. In complete remission, all of the following cell subpopulations collected from peripheral blood (i.e., CD^56+^ NK cells, CD^19+^ B lymphocytes, CD^14+^ monocytes, CD^66+^ granulocytes, CD^34+^ CD^45^ - low blasts), and (i.e., CD^34+^ CD^38−^ CD^123+^ and aldehyde dehydrogenase (ALDH) intermediate leukemic stem cells (LSCs) or CD^34+^ CD^38−^ CD^123+^ and ALDH high hematopoietic stem cells (HSCs)) isolated from bone marrow harbored *DNMT3A* mutations but none of the molecular abnormalities (i.e., *NPM1, IDH1/2* and *FLT3-ITD* mutations) identified at AML diagnosis. Interestingly, *DNMT3A* mutations were not found in CD^3+^ T lymphocytes of these patients (Table 3).

**Table 3:** Molecular abnormalities for the 3 patients who harbored *DNMT3A* mutations at complete remission

UPN	Sample type	Mutation at AML diagnosis	Cell fraction analyzed	*FLT3*	*NPM1*	*DNMT3A*
14	blood	*DNMT3A* R882C	total	ND	ND	+
	BM		total			+
	skin		total			–
	blood		CD3			–
	blood		CD19			+
	blood		CD56			+
	blood		CD14			+
	blood		CD66			+
	blood		CD34			+
	BM		CD34+CD 38-CD123- ALDH high			+
	BM		CD34+CD 38-CD123- ALDH int			+
29	blood	*FLT3-TKD, NPM1A, DNMT3A* R882C	total	–	–	+
	BM		total			+
	skin		total			–
	blood		CD3			–
	blood		CD19			+
	blood		CD56			+
	blood		CD14			+
	blood		CD66			+
	blood		CD34			+
	BM		CD34+CD38-CD123+ ALDH high			+
	BM		CD34+CD 38-CD123+ ALDH int			+
31	blood	*FLT3-ITD, NPM1A, DNMT3A* R882C	total	ND	ND	+
	BM		total			+
	skin		total			–
	blood		CD3			–
	blood		CD19			+
	blood		CD56			+
	blood		CD14			+
	blood		CD66			+
	blood		CD34			+
	BM		CD34+CD38-CD123+ ALDH high	–	–	+
	BM		CD34+CD 38-CD123+ ALDH int			+

For each patient, the mutations that were found at diagnosis (column 3) were analyzed in the following fractions: blood, BM and skin (column 2). Column 4 provides details on the different subpopulations analyzed in the BM, the CD34+ CD38- CD123+ ALDH intermediate and the CD34+ CD38- CD123- ALDH high cells were considered enriched in leukemia-initiating cells and normal hematopoietic stem cells, respectively. Abbreviation: BM, bone marrow; ND, not determined; UPN, unique patient number.

## DISCUSSION

Our data suggest that the use of NGS to monitor MRD based on *IDH1/2* mutations is feasible and effective, as this method enabled us to predict relapse in the majority of patients, with an area under the curve of 0,7971 (95% CI: 0,6693 – 0,9250), and a success rate of 100% if we include the patient who developed MDS. In our cohort *IDH1/2* mutations-based MRD better predicted relapse than *NPM1* mutations-based MRD.

*IDH1/2* mutated patients may benefit from new targeted therapies with specific molecules inhibiting of *IDH1* or *IDH2* mutant proteins [15–18].

Of these targeted inhibitors, 3734 AG-120 is effective at lowering 2-HG levels and restoring cellular differentiation in primary AML cells. This therapy could be more personalized by monitoring *IDH1/2* mutation levels by NGS during and after treatment.

In contrast, *DNMT3A* mutations were not a suitable markers for MRD monitoring because of the persistence of a preleukemic clone carrying *DNMT3A* mutations in 40% of the patients who were in complete remission after a median follow-up of 1439 days (range: 1154–2637 days) which likely reflects the intraclonal molecular heterogeneity of hematopoiesis. Changes in gene mutation frequency were reported between AML diagnosis and relapse, and the expansion of a subclone initially present at a low frequency at the time of diagnosis has been observed at relapse [19].

Our results of the sorted cell populations confirm the molecular heterogeneity of hematopoietic clones at complete remission Liran et al. [20] also reported the presence of *DNMT3A* mutations at a high allelic frequency in highly purified HSCs, progenitors and mature blood cell fractions in AML patients in complete remission but did not observe concurrent *NPM1* mutations, present in the blast cells at AML diagnosis. *DNMT3A* mutant HSCs showed a multi-lineage repopulation advantage over the non-mutated HSCs in xenograft experiments, which suggests that these cells were pre-leukemic HSCs [21]. Altogether, these data suggest that *DNMT3A* mutations could induce “pre-leukemic” abnormal hematopoiesis but remain insufficient for leukemogenesis.

We were not able to perform the mutation screening in samples collected before the diagnosis of AML in our patient population to determine whether *DNMT3A* mutations may have preexisted in the patients whose samples showed a discrepancy between *DNMT3A* and *NPM1* mutation MRD levels. However, the observed *DNMT3A* variant allele frequency in the patients who were in complete remission after chemotherapy ranged from 5 to 45% at post-induction and increased during follow-up, unlike other MRD markers.

Although we could not evaluate whether mutated *DNMT3A* was present in HSCs before AML diagnosis, 3 groups independently reported the emergence of neoplastic blood cell clones with aging [22–24]. Jaiswal et al. reported that *DNMT3A* mutation were the most frequent mutations observed with aging and that patients with *DNMT3A* mutations had a 10- to 50-fold higher propensity for developing hematologic cancer [24]. Similarly, Genovese et al. reported that the frequency of mutations among individuals older than 65 years was 10% and that the most frequent mutations affected *DNMT3A* gene [23]. They also reported that *DNMT3A* mutation was associated with increased risk for developing hematologic cancer that was related to the earlier clone. Our patients were not over 65 years of age and demonstrated an elevated VAF during the CR stage that was higher than the level observed with aging, which suggests that the mutant HSCs were resistant to chemotherapy. How these abnormal hematopoietic clones will be involved in relapse or in the occurrence of new hematological malignancies should be monitored on a long term period.

## PATIENTS AND METHODS

### Patients and samples

This retrospective study included 94 samples from 31 *NPM1* mutation-positive patients (23–70 years old; median: 60 yrs) who were newly diagnosed with AML from the Acute Leukemia French Association (ALFA) - 0701 trial.

### Molecular analysis

*NPM1* mutation monitoring by RTqPCR was performed as previously described [25]. *IDH1/2* and *DNMT3A* mutation monitoring was performed by NGS using an Ion Torrent ProtonTM instrument (life technologies). To obtain very high coverage (i.e., approximately 2 million reads per sample), 24 samples were analyzed per run. Bioinformatic analysis was performed as described in our previous work [26].

Samples from three patients in CR who had persistent *DNMT3A* mutations but no other abnormalities were more extensively investigated. *DNMT3A* quantification by NGS was performed on the following samples: skin, whole peripheral blood, whole bone marrow and blood subpopulations.

### Flow cytometry

The following blood subpopulations were sorted using positive selection immuno-bead kits (Easy Sep Stem Cell): Neutrophils targeted by CD66b antibodies (Ab), monocytes targeted by CD14 Ab, T lymphocytes targeted by CD3 Ab, B lymphocytes targeted by CD19 Ab and NK cells targeted by CD56 Ab. The bone marrow subpopulations that were notably enriched in leukemia-initiating cells included CD34+, CD38-, CD123+, and ALDH intermediate cells. These cells were sorted using a FACS ARIA Sorp based on the membrane expression levels of CD34, CD38, and CD123 and the level of ALDH activity [27].

## CONCLUSION

The NGS technique is an effective tool to monitor MRD in AML patients, but choosing the appropriate MRD markers is crucial to avoid results that are not related to the disease status. Altogether, our findings show that DNMT3A mutation does not participate to relapse or leukemia progression during our period of clinical follow up. Screening of leukemia-initiating mutations, such as *DNMT3A, NPM1* or *IDH1/2* mutations should be performed at diagnosis but only *NPM1* and *IDH1/2* are robust target for MRD monitoring.
